# Assessment and management of radiotherapy beam intersections with the treatment couch

**DOI:** 10.1120/jacmp.v11i2.3171

**Published:** 2010-04-19

**Authors:** Monique van Prooijen, Thilakshan Kanesalingam, Mohammad K. Islam, Robert K. Heaton

**Affiliations:** ^1^ Radiation Medicine Program Princess Margaret Hospital Toronto Ontario Canada; ^2^ Department of Radiation Oncology University of Toronto Toronto Ontario Canada

**Keywords:** beam attenuation, treatment couch, treatment planning, IMRT

## Abstract

The impact of the treatment couch on a radiotherapy plan is rarely fully assessed during the treatment planning process. Incorporating a couch model into the treatment planning system (TPS) enables the planner to avoid or dosimetrically evaluate beam‐couch intersections. In this work, we demonstrate how existing TPS tools can be used to establish this capability and assess the accuracy and effectiveness of the system through dose measurements and planning studies. Such capabilities may be particularly relevant for the planning of arc therapies.

Treatment couch top models were introduced into a TPS by fusing their CT image sets with the patient CT dataset. Regions of interest characterizing couch elements were then imported and assigned appropriate densities in the TPS. Measurements in phantom agreed with TPS calculations to within 2% dose and 1° gantry rotation. To clinically validate the model, a retrospective study was performed on patient plans that posed difficulties in beam‐couch intersection during setup. Beam‐couch intersection caused up to a 3% reduction in PTV coverage, defined by the 95% of the prescribed dose, and up to a 1% reduction in mean CTV coverage. Dose compensation strategies for IMRT treatments with beams passing through couch elements were investigated using a four‐field IMRT plan with three beams passing through couch elements. In this study, ignoring couch effects resulted in point dose reductions of 8±3%.

A methodology for incorporating detailed couch characteristics into a TPS has been established and explored. The method can be used to predict beam‐couch intersections during planning, potentially eliminating the need for pretreatment appointments. Alternatively, if a beam‐couch intersection problem arises, the impact of the couch can be assessed on a case‐by‐case basis and a clinical decision made based on full dosimetric information.

PACS numbers: 87.53.Bn;87.55.Gh;87.55.de;87.56.nk

## I. INTRODUCTION

Radiation therapy techniques are becoming more complex at the same time as they are becoming more precise. There is, therefore, a growing need to visualize the couch elements during the planning process. Couch visualization would ensure that oblique beams avoid high‐density components without resorting to patient trial setups, thereby saving valuable time on the treatment unit and reducing replanning time. Incorporating couch information in treatment plans for rotational therapy may also be beneficial.

Early investigators concentrated on ensuring couch avoidance and basic characterization of treatment couch top attenuation.^(1)^ ^,^ ^(2)^ ^,^ ^(3)^ Muthuswamy^(3)^ reports on a general analytical equation that could be used to determine whether a beam would intersect components of the treatment couch, while De Ost et al.^(1)^ and Meara et al.^(2)^ were concerned with identifying significant increases in skin dose with otherwise negligible attenuation for beam angles of 180°. On this same topic, Meydanci and Kemikler^(4)^ published a more detailed study of increased skin dose due to a carbon fiber tabletop for both 6 and 18 MV photon beams. Vieira et al.^(5)^ published one of the first papers in which inclusion of the couch in the TPS was considered. Finding large attenuations for some components of the Varian Exact Couch, they suggested that visualization of the couch could facilitate the choice of beam angles to avoid these couch parts and, if needed, patient‐specific tolerances on couch position. McCormack et al.^(6)^ attempted to account for lateral couch positioning using a correction factor, but cautioned this could not be used indiscriminately on all plans. More recently, Njeh et al.^(7)^ used a similar strategy for the evaluation of dose delivered by IMRT and found expected dose reductions of 1.6% and 3% for a seven‐field prostate and a nine‐field head and neck plan, respectively.

A number of authors have recommended using the treatment couch top in the TPS, either because of highly attenuating components^(^ ^5^ ^,^ ^6^ ^,^ ^8^ ^,^ ^9^ ^,^ ^10^ ^,^ ^11^ ^,^ ^12^ ^)^ and/or because of concerns about increases in skin dose.^(10)^ ^,^ ^(13)^ ^,^ ^(14)^ Munjal et al.^(8)^ showed that accounting for support structures significantly reduced differences between planned and delivered dose. Myint et al.^(9)^ and Mihaylov et al.^(12)^ showed that differences between planned and measured dose could be reduced to less than 2% if the couch was included in the TPS.

Inclusion of the treatment couch in a TPS has been investigated by Spezi et al.^(10)^ ^,^ ^(11)^ ^,^ ^(15)^ and Mihaylov et al.^(12)^ using a number of different methods. Both find good agreement between planned and measured doses when the couch is included in the plan. Spezi et al.^(11)^ found that including the couch significantly changes IMRT beam parameters in unexpected ways. However, Mihaylov et al.^(12)^ surmised that the influence of the couch in multiple beam plans is likely to be clinically insignificant. This is not unreasonable given that common IMRT techniques use seven or nine beams. In the case of the Varian IGRT couch, the exact position of the couch was found to be unimportant because of its high uniformity; however, inclusion of the couch in the TPS avoided clinically significant dosimetric discrepancies.^(16)^

In this work, we describe our findings for couch attenuation for the Varian Exact Couch (Varian Medical Systems, Palo Alto, CA) and the Sinmed Mastercouch (CIVCO Medical Solutions, Kalona, IA), two couch tops which have not been extensively investigated but which are often installed with accelerators used for treating with arc therapies such as VMAT and RapidArc (Varian Medical Systems, Palo Alto, CA). The data reported are preliminary to an assessment of the dosimetric effect these couches might have on such arc therapy plans. We also illustrate a clinical process to incorporate the treatment couch into any patient plan, replacing the CT‐scanner couch and allowing otherwise normal planning, all using regular planning tools. Our method allows detailed assessment of the impact of the treatment couch on patient dosimetry.

## II. MATERIALS AND METHODS

Our method is based on an in‐house developed process of fusing couch top CT information with the patient CT dataset. Planning system fusion tools were used to transfer contours from the couch dataset to the patient dataset to reflect the treatment geometry in the plan. A density override tool was used to incorporate couch effects in the dosimetry.

**Figure 1:**
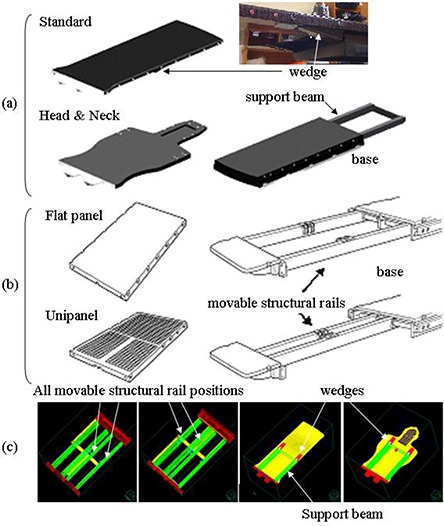
The Mastercouch system (a) with images adapted from Sinmed Mastercouch Elekta Precise User Manual Version 8, CIVCO Medical Solutions; the Exact Couch system (b) with images adapted from the Exact Couch User Guide/Maintenance Manual, Varian Medical Systems; couch model elements (c) for the Exact Couch (first 2 panels) and Mastercouch (last 2 panels). All 4 rail positions of the Exact Couch are shown; in a clinical model, only 2 rails would be present.

Two linear accelerator systems, Elekta Synergy (Elekta, United Kingdom) and Varian Clinac (Varian Medical Systems, U.S.A.), were used in this investigation. The Elekta treatment unit is equipped with the Sinmed Mastercouch (Fig. 1(a). It includes two primary couch top modules, the “Standard” and “Head & Neck”, both of which rest on a support beam and have mating wedge supports to transfer torsion load. The wedge supports on these modules are of a higher density than the rest of the material; their locations are indicated in Fig. 1. The Varian Clinac is equipped with the Exact Couch system (Fig. 1(b) and also has two primary couch top modules, the “Unipanel” and “Flat Panel”. These panels are supported by a structural base consisting of two movable rails that can slide laterally.

All portable couch components, as well as an IMRT phantom, were scanned using a Philips Brilliance CT Big Bore (Philips Healthcare, The Netherlands) in multiple orientations to enable orientation‐specific couch models. Image sets were imported into the Pinnacle TPS (Philips, v 8.0h), with all dose calculations set to the adaptive convolution algorithm using a 2.5 mm calculation grid spacing and a patient air threshold of 0.1 g/cc. All measurements were obtained using a cylindrically‐shaped solid water phantom, having a diameter of 20 cm. Dose was measured using a Keithley 35614 or Fluke 35040 dosimeter and a NE 2571 ion chamber located at the center of the phantom at the linac isocenter. In the TPS, the ion chamber was represented by an ion chamber Region of Interest (ROI) at the center of the phantom. A $10\times 10\;{\text{cm}}^{2}$ 6 MV photon beam with 150 MU exposures was used throughout. For the Sinmed couch top, the effect of higher energies was also investigated.

### A. Addition of the treatment couch to the patient image set

Patient CT scans include the CT unit couch top, which must be removed in the treatment planning system to avoid incorrect dose computation. Within the TPS, the CT couch is replaced with a complete description of the treatment couch top represented as a set of contours. This enables the selection of optimal beam angles in the TPS through the visual display of beam intersections with couch elements. Using treatment couch CT scans, engineering drawings and physical measurements, the relative positions of treatment couch components were drawn as ROI's in the treatment planning system. Each couch top consequently had one corresponding image set and a set of ROI's for each possible orientation, to represent the various components of that specific module (Fig. 1(c).

The support beam of the Sinmed Mastercouch, the only couch part which could not be CT‐scanned, was characterized as a solid component with a uniform density, although the physical structure likely contains a high‐density shell surrounding a low‐density foam core. ROI's for the movable structural rails of the Exact Couch were created at their inside and outside travel positions, providing for four independent rails that could be activated to obtain all possible standard arrangements. Couch image sets and ROI's were exported to a permanent storage directory for simple and efficient retrieval.

The primary image set, of a patient or a phantom, was used for dose calculation and the relevant treatment couch top was assigned as the secondary image set. The Syntegra fusion tool within the TPS was used to position the treatment couch image set with respect to the primary image set. For this purpose, the fusion tool is used in manual mode showing both image data sets, with positioning established in the 3D view and/or the cross‐sectional views. Since the couch information is in a fused secondary image dataset, the couch CT image does not appear in the primary dataset and information about the couch needs to be separately introduced to the primary dataset to be visualized and taken into account during dose calculations. Regions of interest associated with the couch top in the secondary image set were therefore transferred to the primary image set for visualization. Measurement‐derived densities were assigned to enable inclusion in the dose calculations. Differences between CT‐ and measurement‐derived densities ranged from −16% to +129% over a density range of 0.60 to $1.40\;g/{\text{cm}}^{3}$.

### B. Couch model verification

A number of phantom setups were planned in the TPS and reproduced on a treatment unit to verify the accuracy of the geometric relationship between couch elements. At the treatment unit, before commencing our detailed attenuation measurements, one edge of a $10\times 10\;{\text{cm}}^{2}$ light field was used to validate the accuracy of the projected beam angles, consistent with standard visual checks performed by the treatment therapists. Similar geometric integrity tests were performed as part of each subsequent experimental setup.

Each couch element ROI was assigned an initial density based on its respective couch component CT number, which was then fine‐tuned to conform to experimental attenuation data by means of an iterative process in the TPS. In the case of the support beam for the Sinmed Mastercouch, the density was assigned as an average density to the entire structure based entirely on experimental data (due to the inability to perform a CT scan of the structure). We have therefore ignored any internal structure of this component.

The precedence of density assignment for overlapping ROIs in the Pinnacle TPS is determined by their order of occurrence in the ROI list, with the last ROI having precedence. Thus, a large ROI representing air ($0g/{\text{cm}}^{3}$) was always first in the ROI list and was used to override all densities of the CT unit couch top included in patient CT images. The treatment couch top ROI's were then stacked in front of this “air” contour. In standard planning, the Couch Removal Tool in Pinnacle is used to remove CT couch information for dose computation purposes. For this study, it was placed directly below the treatment couch top contours in a technique similar to that of Mihaylov et al.^(12)^ and Spezi et al.^(11)^

A series of experiments were performed to establish the accuracy of the couch model. Each experimental setup was first planned in the TPS and then replicated at the accelerator in order to mimic clinical use. Dose measurements were compared with the TPS dose volume histogram mean of the ion chamber ROI. Results are presented as calculated and measured attenuations relative to values from a reference beam that did not intersect any couch components. For both couch systems, dose measurements were taken for beams passing through the most highly attenuating couch components: the movable structural rails for the Exact Couch and the wedge supports for the Sinmed Mastercouch.

### C. Clinical evaluation

#### C.1 Retrospective clinical study

A retrospective study was executed for five patient plans that presented setup difficulties due to beam‐couch intersection to assess whether such difficulties could be foreseen and thereby prevented. For each clinical plan, the appropriate treatment couch was imported into the TPS and positioned as accurately as possible relative to the patient image using therapy treatment notes to recreate the geometry encountered on the treatment unit. The dosimetric impact of treating through the couch was assessed by comparing changes in the mean CTV dose and volume of PTV covered by 95% of the prescribed dose in the original plan to the same parameters in a plan incorporating the couch model.

#### C.2 IMRT study

In some IMRT plans, treatment through couch elements may be necessary. A study to determine the accuracy of dose calculation for the effects of the couch using this model was done using a cylindrical phantom. Our IMRT test plan consisted of four beams, three of which intersected couch components in some way for a more pronounced effect of the couch in the dose distribution. Dosimetric information was captured using Kodak EDR2 film (Carestream Health, Rochester, NY) at the center of the phantom in the coronal plane. A representative line across the middle of the film was chosen and read with a Macbeth densitometer at 24 equally‐spaced points. A reference measurement for the plans with no couch interference was obtained by delivering all beams passing through the couch with a 180° gantry angle offset. The phantom was similarly rotated when treating with these beams. Active dose compensation was investigated through a third measurement of a plan in which IMRT dose optimization included compensation for the presence of the couch.

## III. RESULTS

The evaluation of beam intersection with couch components using the edge of the light field showed good agreement between the TPS and setup at the treatment unit. The field edge intersected a couch component within $\pm 1^{0}$ of the predicted gantry position.

The attenuations for the Unipanel, Flat Panel, Standard and Head & Neck modules are displayed in Tables 1 and 2. The highest density components can attenuate the beam by between 10% and 20% for a standard 6 MV beam. The average difference between measured and predicted attenuation within a couch top module was between −0.4% and 0.7%. The largest differences were 2.3% and 2.0%, which occurred for beams directed through the rail and/or frame (dense areas) of the Unipanel. These results are consistent with other published results.^(9)^ ^,^ ^(11)^ ^,^ ^(12)^

**Table 1:** Attenuation comparisons of TPS and linac measurements for Exact Couch components at 6 MV.

A: Unipanel Couch Module			
*Description*	*TPS Attenuation (%)*	*Measured Attenuation (%)*	*Difference (%)*
Central Spine Down w/ Both Rails Out:			
Through Unipanel (grid)	0.4	‐0.1	‐0.5
Missing Rail (Lt)	0.9	0.6	‐0.3
Through Rail (Rt) & Unipanel(frame)	8.0	10.3	2.3
Central Spine Up w/ Both Rails In:			
Missing Rail (Rt)	0.3	1.0	0.7
Through Unipanel (frame)	2.7	4.7	2.0
Through Rail(Lt) & Unipanel(frame)	14.8	15.3	0.5
Central Spine Up w/ Right Rail In & Left Rail Out:			
Through Unipanel & Rail (Rt)	16.7	17.4	0.7
Through Unipanel, Missing Rail (Rt)	4.6	4.2	‐0.4
B: Flat Panel Couch Module			
*Description*	*TPS Attenuation (%)*	*Measured Attenuation (%)*	*Difference (%)*
Right Rail Out & Left Rail In:			
Through Rail (Lt) & Flat Panel	13.6	13.5	0.0
Missing Rail (Lt), Through Flat Panel	2.1	2.2	0.1
Through Rail (Rt) & Flat Panel	8.4	7.8	‐0.6
Both Rails Out:			
Through Flat Panel	2.2	2.1	‐0.1

**Table 2:** Attenuation comparisons of linac and TPS measurements for Mastercouch components.

A: Standard Couch Module.				
*Description*	*Photon Beam Energy (MV)*	*TPS Attenuation (%)*	*Measured Attenuation (%)*	*Difference (%)*
Through Module:				
	6	1.8	1.9	0.1
	10	1.3	1.4	0.1
	18	1.1	1.1	0.0
Through Module (Rt side):				
	6	2.6	3.1	0.5
	10	2.0	2.0	0.3
	18	1.7	1.7	0.0
Through Wedge (Rt):				
	6	15.5	14.5	‐1.0
	10	12.8	11.9	‐0.9
	18	11.0	9.8	‐1.2
ThroughMissing Wedge (Lt):				
	6	3.2	2.2	−1.0
	10	2.6	1.9	−0.7
	18	2.2	1.5	−0.7
Through Support Beam^a^ (Lt side):				
	6	6.8	5.9	−0.9
	10	5.4	4.7	−0.7
	18	4.7	3.7	−1.0
B: Head & Neck Couch Module.				
*Description*	*Photon Beam Energy (MV)*	*TPS Attenuation (%)*	*Measured Attenuation (%)*	*Difference (%)*
Through Module:				
	6	2.5	2.6	0.1
	10	2.1	2.2	0.1
	18	1.7	1.6	−0.1
Through Support Beam^a^ (Rt side):				
	6	6.6	6.6	0.0
	10	5.5	5.5	0.0
	18	4.7	4.3	−0.4
Through Wedge (Rt):				
	6	18.1	16.7	−1.4
	10	15.0	13.9	−1.1
	18	12.9	11.5	−1.4

a unscanned component

**Figure 2:**
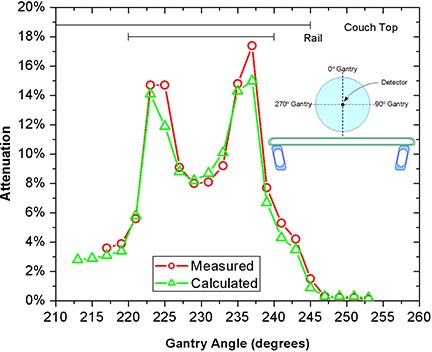
Predicted and measured attenuation for the movable structural rail and the Flat Panel of the Exact Couch.

**Figure 3:**
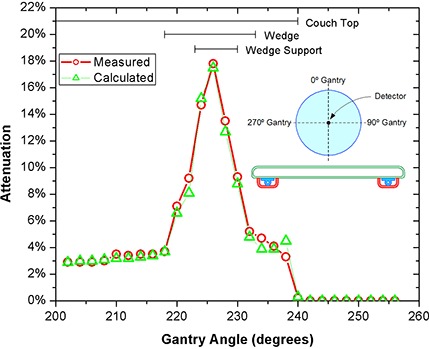
Predicted and measured attenuation for the wedge structure of the Mastercouch using the Standard module.

Figure 2 shows dose attenuation values when treating through the densest couch components of the Exact Couch: the rail and Flat Panel. Results of a similar experiment performed on the Sinmed Mastercouch, treating through the thick part of the wedge‐shaped structure on the Standard module, are presented in Fig. 3.

Results for the retrospective patient plan study are presented in Table 3. In this set of plans, the addition of couch contours resulted in a maximum decrease in PTV coverage by the 95% isodose volume of just over 3%. The differences in CTV mean dose ranged from 0.1% to 1.3%.

**Table 3:** Retrospective patient plan analysis to assess the impact of the treatment couch on dose distribution.

Patient	*PTV* ^a^ *(Without Couch) (%)*	*PTV* ^a^ *(With Couch) (%)*	*Δ Volume (%)*	*CTV* ^b^ *(Without Couch) (cGy)*	*CTV* ^b^ *(With Couch) (cGy)*	*Δ Dose (%)*
1	99.1	98.6	0.5	3592	3590	0.1
2	99.9	99.9	0.0	7779	7752	0.4
3	90.8	87.5	3.3	2993	2971	0.7
4	98.7	96.8	1.9	5042	4978	1.3
5	95.7	94.9	0.8	4264	4225	0.9

a volume covered by 95% of prescribed dose

b mean dose

**Figure 4:**
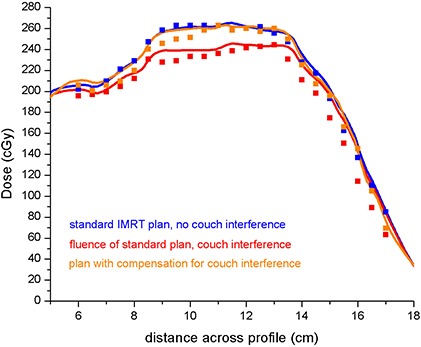
IMRT film experiment results. — Pinnacle, ■ film measurement. Data show one line in a coronal cross‐section of the treatment plan where the effect of the couch was clearly seen.

Figure 4 shows a profile across the center of the coronal dose distribution for the IMRT attenuation compensation test. A standard plan in which no couch components are considered in planning and are not an issue during treatment is compared to the same plan with couch components in the path of the beam, and to a third plan in which the field intensity distributions compensate for the presence of the couch components. The first case demonstrated the ideal treatment setup and corresponding distribution; the second indicated what would occur if couch components were allowed to interfere with treatment delivery; and the third validated the accuracy of dose compensation from the proposed method of couch characterization in the TPS. Based on the Macbeth readings and relative to the unattenuated reference plan, delivery through the couch shows an average point dose deviation from the standard plan of 21.1±6.6 cGy, or about 8% of the intended dose (260 cGy). However, for the plan optimized with the couch densities, the distribution becomes similar to that of the reference plan, with a measured average dose deviation of 5.7±4.5 cGy, or about 2% from the reference plan.

## IV. DISCUSSION

The process we envision for using this model is summarized below in order of increasing degree of intervention.

1. In some plans, chosen beam angles are known to be potentially problematic with respect to treating through highly attenuating couch elements. These plans require an extra patient visit to determine if a problem indeed exists. The first aim of our process, therefore, is to be able to determine, at the planning stage, whether beams will intersect with attenuating couch elements. We reviewed five clinical cases in which setup difficulties had been encountered. In each case, a positioning adjustment was required to avoid beam attenuation. Had the couch model been used during the initial planning stage, the setup problems could have been identified and perhaps avoided through a modification of the initial beam angles. After gaining sufficient confidence with this method, it may be possible to eliminate pretreatment setups, thereby reducing the load on treatment units and requiring fewer patient visits.
2. If a problem is encountered during a first treatment visit, the model can be used to find more appropriate beam angles. A replan can then be initiated with confidence.
3. If other beam angles are not optimal, the couch model can be used to simulate the treatment and determine the impact of the couch on the dose distribution. This may occur more frequently with the implementation of arc therapy treatments. Decisions for further action can then be based on an accurate assessment of the dosimetry.
4. In cases where accurate two‐dimensional repositioning relative to the couch is available, one may consider compensating for couch elements in the beam using IMRT if the dosimetry warrants such an intervention. Our IMRT test showed compensation for dose reduction due to beam attenuation by couch elements can be successfully incorporated into individual patient treatment plans.

A full clinical implementation of this process requires some refinement of patient setup procedures. The accuracy of the results shown in Figs. 2 and 3 clearly demonstrates both the validity of the geometrical representation of couch contours and the accuracy of the dose predicted by the TPS, and highlights the need for accurate positioning relative to the couch elements. To achieve the same accuracy in patient positioning may require indexed immobilization devices to constrain setup variation, both longitudinally and laterally. Conversely, dose compensation demands accurate localization of the patient; hence without indexed immobilization, avoidance of highly attenuating couch elements is the only credible method available for accurate treatment.

Although we have a detailed description of the couch, the patient CT field of view (FOV) may not be large enough to include the entire couch structure. In the Pinnacle TPS, this means the couch is truncated. In such a case, important dosimetric information about the couch and its extent may be lost. For clinical implementation, therefore, the use of the model during planning must be considered at the time of patient scanning, so that an appropriate FOV can be used. A similar problem would arise if the model were extended to predict gantry collisions. This would require the introduction of an exclusion zone, which could also extend beyond the FOV used at the CT.

The couch model is a hybrid of CT‐determined geometric information and average physical densities deduced from dosimetric attenuation measurements. For those components that could not be scanned (viz. Sinmed Mastercouch support beam), geometric information was taken from manufacturer documentation. Calculations using densities obtained directly from the CT‐scanner did not provide results consistent with attenuation measurements for some couch components. Table 4 gives the dosimetrically consistent physical densities in comparison to CT scanner‐derived densities and those suggested by the manufacturer. While some of the differences seen are due to the treatment of heterogeneous density regions as homogeneous elements, part of the difference can be attributed to the underlying assumptions of the HU to density conversion. Treatment planning systems assign a density based on the assumption of tissue‐like composition,^(17)^ and consequently the HU of non‐tissue materials may be mapped onto an inappropriate density. Thus, for the purpose of dosimetry, a fine‐tuning of the densities on the basis of detailed attenuation measurements is required.

**Table 4:** Densities of couch components of the Exact Couch and Sinmed Mastercouch.

*Structure*	*Assigned Density (g/cm^3^)*	*CT Image Density (g/cm^3^)*	*Manufacturer Reported Density (g/cm^3^)*
Exact Couch:			
Flat Panel	0.53	0.12–0.47
Unipanel	0.72	1.15–1.18	0.8^a^
Movable Structural Rails	1.15	1.28	1.5–1.7^a^
Sinmed Mastercouch:			
Standard	0.60	0.06–1.0	0.5^b^
Head & Neck	1.00	0.07–1.25
Support Beam	0.15	not scanned
Wedge	0.48	0.8–1.0
Wedge Support	1.40	1.2–1.3

a Obtained by dividing manufacturer's reported area density by observed non‐air thickness in couch part cross section.

b Obtained using Pinnacle density override to comply with attenuation reported by manufacturer.

To visualize the couch structures in the primary image dataset, the couch ROI's need to be copied from their high resolution representation to a lower resolution patient image set. This results in somewhat less well‐defined couch edges and may create an error on the order of 1 or 2 pixels or 1.5 to 3 mm. On very small‐dimensioned parts, this may have a measurable effect on the dosimetry. Using the attenuation coefficient for carbon fiber (obtained by Munjal et al.^(8)^), $0.082{\text{cm}}^{-1}$, the expected attenuation over a distance of 1.5 mm, 1 pixel, is about 3%. Therefore, some of the error shown in Tables 1 and 2 may be due to transferring couch ROI's from one resolution to another.

## V. CONCLUSIONS

Incorporating the treatment couch into the TPS is feasible and allows assessment of the impact of the treatment couch in a specific plan for its clinical significance on dose distribution. Our process requires minimal additional planning time and can be largely automated. The couch can be repositioned at any stage of the planning process, even after the addition of beams. Patient setup problems involving the couch can be foreseen at the planning stage, potentially eliminating the need for pretreatment appointments. In addition, the model can be used to assess the impact of the couch for specific patient plans and to determine whether or not beam attenuation is clinically significant on a case‐by‐case basis. If the dosimetric impact is significant, inverse planning can be used to compensate for dose reduction.

The work presented was focused on two types of treatment couch that are of particular interest because they are installed on arc therapy‐capable units. Future work will focus on assessments of treatment couch effects during VMAT dose delivery.
